# Safety, effectiveness and tolerability of sublingual ketamine in depression and anxiety: A retrospective study of off-label, at-home use

**DOI:** 10.3389/fpsyt.2022.992624

**Published:** 2022-09-28

**Authors:** Kazi Hassan, William M. Struthers, Aditya Sankarabhotla, Patrick Davis

**Affiliations:** ^1^Nue Life Health, Miami, FL, United States; ^2^Psychology Department, Wheaton College, Wheaton, IL, United States; ^3^Children's Hospital, Boston, MA, United States

**Keywords:** depression, anxiety, ketamine, sublingual, treatment resistant depression (TRD)

## Abstract

Intravenous and intranasal ketamine have been shown to be effective therapeutic options in patients suffering from treatment-resistant depression (TRD). The use of sublingual (SL), rapid dissolve ketamine tablets (RDT) offers a novel approach for delivery for mental health indications. This study assessed the effectiveness and safety of self-administration of off-label, SL, rapid dissolve ketamine tablets (RDT) at-home for depression and anxiety. Intake scores on the Generalized Anxiety Disorder Screener (GAD-7) and Patient Health Questionnaire (PHQ-9) were compared to scores after treatments of three doses of ketamine RDT, and after six doses of ketamine RDT. After three doses of SL ketamine, 47.6% of patients showed a significant decrease in PHQ-9 scores, and 47.6% of patients showed a significant reduction in GAD-7 scores. Reduction rates were higher in those patients who completed a clinically recommended six doses of RDT ketamine. This study demonstrates that SL ketamine is a novel, safe, and effective treatment for TRD and treatment-resistant anxiety. SL ketamine offers an alternative therapeutic approach to IV ketamine when treating those with TRD.

## Introduction

Major Depressive disorder (MDD) is a common and debilitating psychiatric condition that affects nearly 350 million people globally (1). Approximately 8.1% of Americans aged 20 and over reported depression symptoms in a given 2-weeks period (2). First-line treatment of MDD utilizes antidepressants that prove to be effective in alleviating symptoms in approximately 50% of patients (3). It is estimated 30% of MDD patients fail to respond to at least two antidepressants resulting in treatment-resistant depression (TRD). Suicide risk for TRD patients is higher than that of non-TRD patients diagnosed (4). Range of symptom severity, sequelae, and clinical comorbidity continue to be an area of interest when determining best course of treatment for MDD.

Clinicians have also long recognized that treatment of depression may be unsuccessful if accompanying anxiety disorders are not recognized and addressed (5). Of note, generalized anxiety disorder (GAD) is known to have a high comorbidity rate in patients with TRD (6, 7). Benzodiazepines are commonly utilized in the treatment of depression with anxiety and offer a different pharmacodynamic profile when compared to antidepressants (8), it has been reported that IV ketamine antidepressant effects are interfered with in those concurrently using benzodiazepines (9). The use of benzodiazepines complicates the use of ketamine since anxiety and depression co-occur frequently, and either's symptoms may present as the primary diagnosis. The National Comorbidity Survey demonstrated that an anxiety disorder was comorbid in 58% of the patients diagnosed with MDD at some point in their lifetime (10). These comorbid anxiety symptoms (and potential comorbidity) can complicate the treatment of depression and comorbid anxiety is associated with a greater severity of depressive symptoms and an increased time to recovery. Comorbid anxiety is also associated with a resistance to pharmacological treatment for depression (i.e., TRD), increased incidence of relapse, and suicidal ideation (11). A balanced assessment and approach to TRD also requires an examination of comorbidity issues which may mediate response to treatment.

In recent years there has been increased attention paid to alternative pharmacological interventions for patients with TRD (12). One of these drugs is the anesthetic ketamine (12–16). Ketamine's pharmacodynamic profile is that of a dissociative anesthetic and N-methyl-d-aspartate receptor (NMDA) receptor antagonist (17–19). Berman et al. was the first study to reveal that a single intravenous (IV) dose of ketamine as effective in treating MDD (20). Their randomized, doubl- blind study on a small group of subjects with MDD using IV ketamine hydrochloride (0.5 mg/kg) or saline over 2 days resulted in a significant reduction of depression symptoms as measured by a reduction in Hamilton Depression Rating Scale after ketamine treatment when compared to saline. Reports examining the effectiveness of ketamine in randomized, double-blind controlled trials in the past decade has seen an increase in clinical interest (21, 22). A review of the literature reveals intravenous (IV) infusions of ketamine tend to be the preferred route of administration (23). A systematic review of 288 published studies on IV ketamine's side effects and safety profile reported that acute side-effects were commonly associated with single-dose use ketamine for TRD, though they are generally transient and spontaneously resolve and are relatively understudied (24). A limitation of IV ketamine infusions is that they require a medicalized setting and incur considerable resources and cost. There is sufficient evidence of effectiveness to warrant inclusion as a treatment option with intranasal (IN) esketamine approved by the FDA in 2019 for TRD. Several randomized control trials of racemic ketamine have been published since IN esketamine's approval demonstrating its effectiveness as a tool in treating MDD or TRD (25–36). Studies on transmucosal and sublingual (SL) ketamine provide another alternative to oral ketamine use in the treatment of depression (37–41). Oral ketamine, while less commonly used compared to the gold standard of IV ketamine, has been tested extensively from a pharmacodynamic and pharmacokinetic perspective. Specifically, oral ketamine has been shown to undergo extensive first-pass metabolism and consequently has approximately 10–20% bioavailability, while SL ketamine has a bioavailability of approximately 30% (42, 43), a fact which must be taken into account when designing outpatient oral or SL ketamine treatment regimens. A recently published manuscript has reported similar effectiveness and safety of at-home SL ketamine using a prospective study design (44). The authors found that SL ketamine (three treatments with doses ranging 300–450 mg/kg) in combination with telehealth produced approximately 60% reduction in PHQ-9 and GAD scores at the 4-weeks timepoint compared to baseline scores, roughly comparable with our study. Further work, which we are currently engaged in, will be needed to delineate not only the most effective dosing regimen, but also to identify likely responders based on patient characteristics.

The present study is a retrospective review of patients who had received off-label ketamine using a rapid dissolve tablet for SL delivery treatment at-home for their TRD. The present study examined the effectiveness and safety of the clinical use of at-home, self-administered SL ketamine (300/450 mg) on depression and anxiety symptoms in treatment-resistant patients. This data is related to patients who were treated at home, a model that was created in response to COVID-19 restrictions and using off-label prescription of SL ketamine rapid dissolve tablets, and extends on previous research in that it is the first of its kind examining at-home use of SL ketamine within a clinical context.

## Methods

### Ethics approval

The protocol for this retrospective study was reviewed by the Wheaton College (Wheaton, IL) Institutional Review Board (Protocol #1828103-1; Exempt Category 4.iii) which follows the ethical principles outlined in 45 CFR 46.104(d). The protocol was approved on 11/15/2021.

### Dataset and chart review process

**Figure 1:**
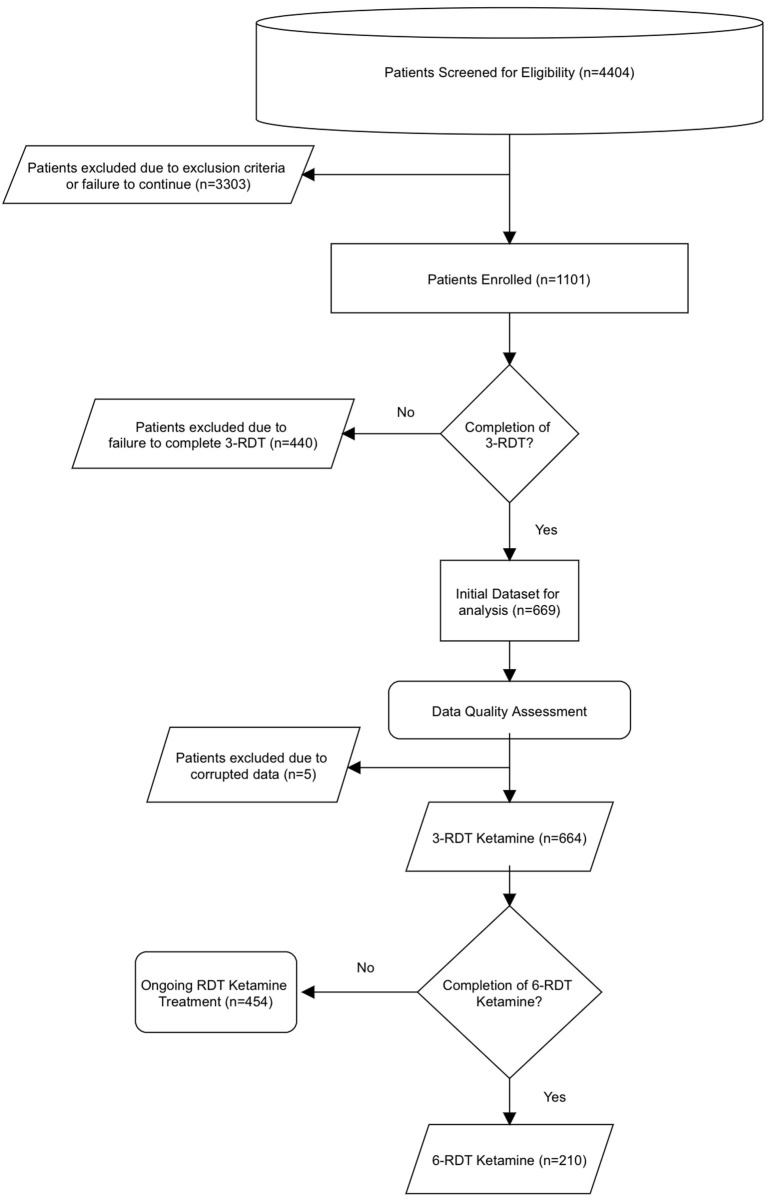
Dataset and process flowchart.

An overview of the dataset analysis and process by which the data was obtained is shown in Figure 1. Data was obtained from a telemedicine practice that offered ketamine treatment that specializes in at-home, SL ketamine use. Between 12/1/2020-9/30/2021, 1101 individuals experiencing treatment-resistant anxiety or depression sought treatment from the telemedicine practice My Ketamine Home. A new patient registration form including a detailed health history and demographic information was completed, and a telemedicine intake consult was then performed. Relevant medical history was evaluated by Psychiatric Mental Health Nurse Practitioners to diagnose MDD and Generalized anxiety on DSM-5 criteria. This intake also included: (1) any current or presenting medical illnesses or previous psychiatric diagnosis, (2) information about previous failed pharmacological treatments for any depression or anxiety, and (3) other comorbid symptoms. Additionally, respondents were given the Patient Health Questionnaire (PHQ-9) to establish an intake baseline for their depressive symptoms, and the Generalized Anxiety Disorder Screener (GAD-7) to determine baseline anxiety symptoms. Based on this information, an evaluation for the clinical appropriateness of ketamine treatment for depression or anxiety was made by the practice. Exclusion criteria for ketamine consisted of indications of any of the following conditions/symptoms: (1) active suicidal ideation, (2) uncontrolled hypertension (HTN), (3) liver disease, or (4) schizophrenia, active substance use disorder, or other primary psychotic disorder. Uncontrolled HTN was defined as systolic blood pressure ≥140 mmHg or an average diastolic blood pressure ≥90 mmHg and patients were excluded based on self-reported BP screening. Absence of liver disease was excluded on the basis of available normal liver function test report information and self-reported past medical history. Potential for diversion for abuse was evaluated by psychiatric mental health nurse practitioners by querying state prescription monitoring databases as part of the medical consultation. Patients who were not excluded were provided with information about the ketamine treatment. Terms and conditions for use of data use were addressed during the onboarding process. Patients then signed a detailed informed consent form.

After obtaining informed consent, an express-mailed package containing a 300 mg rapid dissolve tablet (RDT) of ketamine for SL administration with instructions was shipped to the patient. The basis for a treatment course of 6 treatments was based on the extensive evidence showing effectiveness of 6 IV ketamine infusions and to mirror prior treatment schedules as closely as possible in order to minimize unforeseen variables (45). In line with this, a first shipment contained an RDT tab and additional information providing comprehensive instructions on how to safely to self-administer the ketamine RDT. These instructions included careful preparation and creation of an optimal set and setting (i.e. quiet comfortable environment with dedicated time before and after to meditate and/or reflect), safety instructions including fall risk precautions and avoidance of any sharp or dangerous objects or operation of heavy machinery including cars was explicitly prohibited as was the combination of any other psychotropic substance including alcohol or marijuana. Patients on benzodiazepines were instructed to hold this medication on the day of ketamine therapy due to evidence that it may reduce antidepressant effectiveness (46). This guidance instructed patients to consume the RDT in the presence of a sitter who would provide support as needed. This sitter was also provided ketamine safety education by the medical provider which included information about fall risk and the avoidance of drugs or alcohol. After ketamine use, patients were instructed to recline with an eye-covering (i.e., a sleep mask) in a quiet location. Patients were instructed to listen to provider-prepared music using headphones to minimize distraction and support the ketamine experience.

Upon completion of this initial ketamine administration, patients were asked to complete an online experience report for this first ketamine dose for clinical review by the provider. The decision to use ketamine at 300 mg was determined by providers with experiential CME training in ketamine therapy. This training provided the guidance for determination of the clinically recommended course of ketamine treatment (12). Patients were instructed to self-administer their doses twice a week and complete the online experience survey within 2 days of their final dose from the shipment. The duration of therapy ranged from 2–3 weeks to complete the number of doses prescribed. Decisions to increase doses to 450 mg were made collaboratively between the patient and providers. Based on data from the patient's experience report, a clinical judgment was then made by the provider to maintain or increase (up to 450 mg) the dosage of a second shipment containing two additional ketamine RDT.

A second shipment contained either two 300 mg RDT or two 450 mg RDT, and was also express-mailed to patients. Patients repeated the self-administration procedure for the two additional RDT treatments on separate days, bringing their ketamine treatment to three doses (3-RDT). Upon completion, patients were again asked to complete the online experience report which included the PHQ-9 and GAD-7 measures for clinical review, and rank experiences (None, Mild, Moderate, Severe) of several potential side effects. These included: Anxiety, Blurred Vision, Difficulty Speaking, Difficulty Thinking, Dizziness, Loss of Balance, Memory Issues, Nausea, Pain with Urination, Palpitations, and Pelvic Pain.

After a review of the patient report, a clinical determination for a final shipment of 3 additional ketamine RDT (300 mg or 450 mg) was made, and a third shipment was sent. Patients were again instructed to use the three additional doses as directed and then complete another experience report with PHQ-9, GAD-7, and side effect measures after taking their sixth ketamine RDT (6-RDT) within 2 days of their final dose from the shipment. In summary, data from all patients who had contacted the practice and then completed the follow up surveys after at least 3-RDT ketamine were analyzed. Analysis of the data revealed a subgroup of patients that had received an additional three RDT ketamine (raising their total to six; 6-RDT) that had completed follow up surveys was also conducted.

### Statistical analysis

SPSS (v28) software was used for frequencies and descriptive measures, and to perform inferential analyses of changes in GAD-7 and PHQ-9 scores between intake levels and post-treatment scores. Wilcoxon Matched Pairs analysis were performed for GAD-7 and PHQ-9 scores for both patients at three-, and six-ketamine RDT treatment times as available. Reductions of ≥ 50% of raw score reductions and from intake are also reported, as well % reductions and clinically significant reductions in those patients whose intake GAD-7 and/or PHQ-9 scores placed them in the Moderate to Severe ranges at intake.

## Results

### Sample characteristics

A total of 4,404 people were assessed for eligibility out of which 1,101 patients were enrolled. A review of their clinical data revealed that 669 patients had completed a course of treatment including at least three SL ketamine RDT (3-RDT) and completed the post-treatment experience report. Of these, 5 patients contained missing or corrupted data and were excluded when analyzing GAD-7 and PHQ-9 measures, however their reports of side effects are included in Table 1. Of the remaining 664 patients remaining for GAD-7 and PHQ-9 data analysis 210 patients had received a third shipment with three additional ketamine RDT. These patients completed an additional post-treatment experience report after completing a clinically recommended six ketamine RDT treatment course (6-RDT, n=210). Based on this process we analyzed a group of patients actively involved in ongoing treatment at the time of data collection who had finished 3-RDT of SL ketamine (*n* = 464), and a second group of patients who had completed a 6-RDT course of SL ketamine. Demographic characteristics are shown in Table 2.

**Table 1:** Ketamine side effect incidence and severity after 3- or 6-RDT.

**3-RDT ketamine (*****n*** = **669)**					**6-RDT ketamine (*****n*** = **210)**				
**Side effect**	**None**	**Mild**	**Moderate**	**Severe**	**Side effect**	**None**	**Mild**	**Moderate**	**Severe**
**Anxiety**	72.50%	21.30%	5.16%	1.05%	**Anxiety**	76.42%	19.34%	3.46%	0.79%
**Blurred vision**	45.67%	38.12%	13.53%	2.69%	**Blurred vision**	47.01%	36.01%	13.99%	2.99%
**Difficulty speaking**	59.57%	33.41%	5.90%	1.12%	**Difficulty speaking**	58.81%	31.76%	7.86%	1.57%
**Difficulty thinking**	61.43%	29.82%	7.70%	1.05%	**Difficulty thinking**	60.85%	29.56%	8.33%	1.26%
**Dizziness**	34.98%	43.57%	17.64%	3.81%	**Dizziness**	35.53%	41.98%	19.34%	3.14%
**Loss of balance**	33.33%	43.05%	20.40%	3.21%	**Loss of balance**	37.11%	40.41%	19.18%	3.30%
**Memory issues**	83.18%	13.98%	2.69%	0.15%	**Memory issues**	84.43%	13.36%	3.30%	0.47%
**Nausea**	64.42%	23.09%	9.49%	2.99%	**Nausea**	68.40%	19.81%	8.65%	3.14%
**Pain with urination**	98.21%	1.57%	0.22%	0.00%	**Pain with urination**	97.48%	2.36%	0.16%	0.00%
**Palpitations**	90.21%	7.77%	1.87%	0.15%	**Palpitations**	91.19%	7.86%	0.63%	0.31%
**Pubic pain**	98.21%	1.42%	0.22%	0.15%	**Pubic pain**	98.43%	1.10%	0.31%	0.16%

Percentage of all subjects who reported None, Mild, Moderate, or Severe after their 3^rd^ or 6^th^ RDT of Ketamine.

**Table 2:** Demographic characteristics and GAD-7 and PHQ-9 scores.

		**Age**							
**Gender**	***N* (%)**	** < 20**	**21–30**	**31–40**	**41–50**	**51–60**	**Over 61**	**Missing**	**Age range**
Female	266 (40.7)	4	49	96	64	31	22	3	19-74
Male	288 (44.0)	2	54	128	59	36	9	0	17-82
Not reported/other	110 (15.3)	1	19	42	27	12	6	0	20-70
**Total**	664 (100%)								
**3-RDT ketamine**	***N*** **(%)**	**Intake GAD-7**	**3-RDT GAD-7**		**GAD-7 change**		**≥50%** **Reduction %**		**Clinically significant** **reduction/remission %**	
All patients after 3-RDT	664 (100%)	11.81 ± 5.50		6.94 ± 5.13		4.86 ± 5.18*		47.60%		-	
Moderate to severe	418 (64.9%)	15.29 ± 3.45		8.65 ± 5.40		6.64 ± 5.32*		47.60%		63% / 23.9%	
GAD-7 intake							
	***N*** **(%)**	**Intake PHQ-9**		**3-RDT PHQ-9**		**PHQ-9 change**		**≥50%** **Reduction %**		**Clinically significant** **reduction/remission %**	
All patients after 3-RDT	664 (100%)	13.24 ± 6.04		7.60 ± 5.34		5.64 ± 5.33*		47.60%		-	
Moderate to severe	463 (71.9%)	16.36 ± 4.20		9.13 ± 5.36		7.23 ± 5.33*		49.50%		59% / 20.7%	
PHQ-9 intake							
**6-RDT ketamine**	***N*** **(%)**	**Intake GAD-7**		**6-RDT GAD-7**		**GAD-7 change**		**≥50%** **Reduction %**		**Clinically significant** **reduction/remission %**	
Patients after 6-RDT	210 (100%)	11.74 ± 5.47		5.86 ± 4.87		5.88 ± 5.02*		47.60%		-	
Moderate to severe	133 (63.3%)	15.11 ± 3.61		7.50 ± 5.12		7.61 ± 5.02*		60.20%		69.2% / 33.8%	
GAD-7 intake							
	***N*** **(%)**	**Intake PHQ-9**		**6-RDT PHQ-9**		**PHQ-9 change**		**≥50%** **Reduction %**		**Clinically significant** **reduction/remission %**	
Patients after 6-RDT	210	13.90 ± 6.20		6.60 ± 5.19		7.30 ± 5.85*		61.40%		-	
Moderate to severe	156 (74.2%)	15.76 ± 5.61		7.88 ± 5.61		7.88 ± 5.85*		65.40%		71.2% / 32.7%	
PHQ-9 intake							

The asterisk (*) indicates statistical significance as cited in the text (*p* < 0.001).

### Safety

A review of systems and follow up visits systematically screened for major adverse events (symptoms requiring medical care including hospitalizations as part of structured clinical interview in follow-up). Minor adverse events and side effects including nausea, dizziness, headache, loss of balance, were assessed on self-report questionnaires for each set of experiences. Side effects of ketamine RDT that were reported as part of the experience report are shown in Table 1. These effects were self-limited and resolved without any further medical intervention.

### Impact of three RDT ketamine-induced impact on depression and anxiety

**Figure 2:**
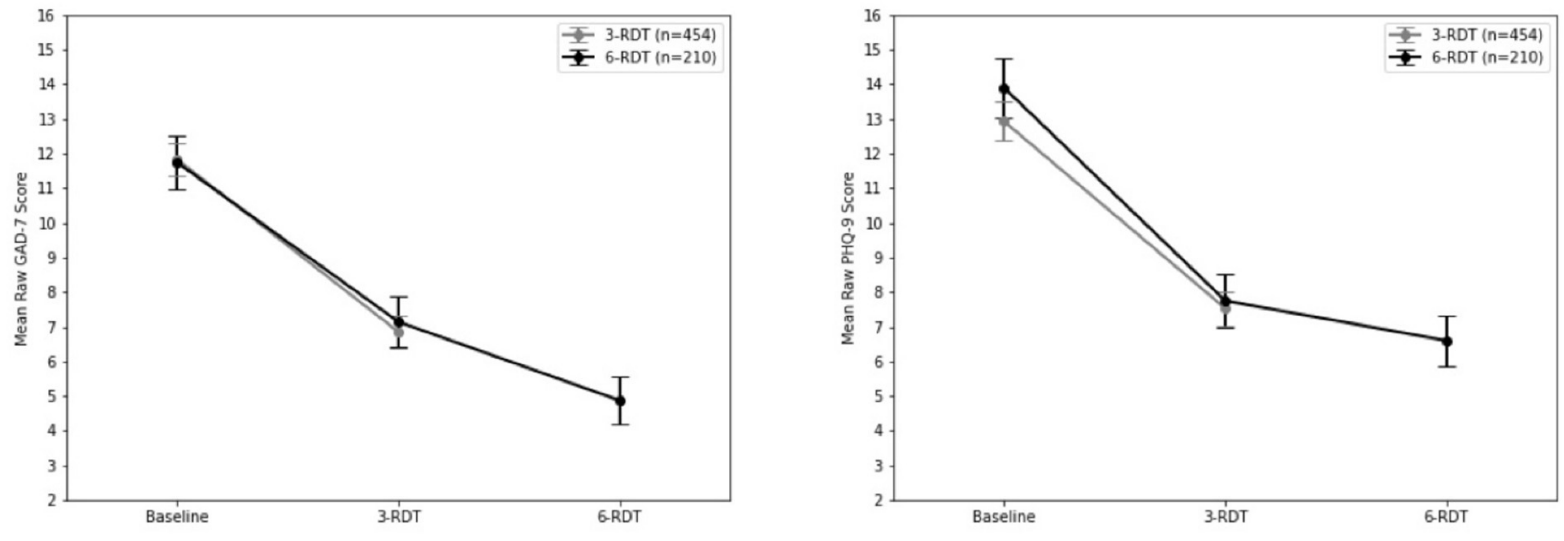
Mean GAD-7 and PHQ-9 scores at baseline, after 3-RTD, and 6-RDT ketamine treatments.

Wilcoxon Matched Pairs analysis of all patients after 3-RDT (*n* = 654) indicated a statistically significant decrease from GAD-7 intake after three ketamine RDT treatment (*z* = −18.52, *p* < 0.001). Average raw GAD-7 score reduction was−4.86, with 47.6% of patients (*n* = 316) having experienced a reduction of 50% or more when compared to their intake GAD-7 score (see Figure 2). As indicated in Table 2, 47.6% (*n* = 199) of patients whose intake GAD-7 scores placed them in the Moderate to Severe range (GAD-7 ≥ 10; *n* = 418) reported reductions to at least half of their intake scores. Of these same Moderate to Severe patients, 63% reported clinically significant drops in GAD-7 scores resulting in either None (0–4) or Mild (5–9) anxiety categorization. The remaining 36.4% (*n* = 152) of Moderate to Severe patients at intake did not experience a clinical reduction in anxiety scores after three ketamine RDT treatment.

Analysis of changes in PHQ-9 depression scores revealed statistically significant reductions after three RDT ketamine treatment (z = −19.71, *p* < 0.001) when compared to intake scores (Table 2). There was an overall reduction of 47.59% from intake to post-ketamine treatment PHQ-9 scores. In patients whose intake PHQ-9 scores placed them in the Moderate to Severe range (*n* = 463), three RDT ketamine treatment resulted in PHQ-9 scores dropping to at least half of intake scores in 49.5% of patients (*n* = 229). Of these same Moderate to Severe PHQ-9 intake patients, 59% (*n* = 273) reported clinically significant drops that placed them in the None (0–4) to Mild (5–9) depression categories. The remaining 41% (*n* = 190) did not experience a clinical reduction in depression scores after three ketamine RDT.

### Impact of six RDT ketamine-induced impact on depression and anxiety

Wilcoxon Matched Pairs analysis of patients who completed a clinically recommended six dose course of RDT ketamine treatment (*n* = 210) revealed a statistically significant decrease in final reported GAD-7 scores when compared to intake after 3-RDT (*z* = −10.12, *p* < 0.001) that was comparable to those reported in the previous group of ongoing patients (see the above 3-RDT). Upon completion of three additional ketamine treatments there was an additional decrease in GAD-7 rank scores (z = −4.62, *p* < 0.001; see Figure 2).

As shown in Table 2, average raw GAD-7 score was reduced by 5.88, and 72.4% of those receiving 6 ketamine RDT (*n* = 152) experienced a reduction of 50% or more from their intake GAD-7 score. For patients whose intake GAD-7 scores placed them in the Moderate to Severe range (see Table 2; *n* = 133), six ketamine RDT treatment resulted in GAD-7 scores dropping to at least half of intake GAD-7 scores in 60.2% (*n* = 80). For these same patients with Moderate to Severe intake GAD-7 scores (*n* = 133; Table 2), 69.2% (*n* = 92) reported clinically significant reductions resulting in None (0–4) or Mild (5–9) post-treatment anxiety categorization. The remaining 30.8% (*n* = 41) of intake Moderate to Severe patients did not experience a clinical reduction in anxiety after six ketamine RDT treatment.

Similarly, Wilcoxon Matched Pairs analysis of PHQ-9 scores revealed a significant reduction in PHQ-9 scores after three RDT ketamine doses (*z* = 10.96, *p* < 0.001) and then after 6-RDT (*z* = 4.45, *p* < 0.001). Of patients whose intake PHQ-9 scores placed them in the Moderate to Severe range (*n* = 156), six ketamine RDT treatment resulted in PHQ-9 scores dropping to at least half of intake scores in 65.4% of patients (*n* = 102). Of these same Moderate to Severe PHQ-9 intake patients, 71.2% (*n* = 111) reported clinically significant drops that placed them in the None (0–4) to Mild (5–9) depression categories. A comparison of 3-RDT only (*n* = 454) and 6-RDT (*n* = 210) GAD-7 and PHQ-9 mean scores from Baseline, 3- and 6-RDT are shown in Figure 2.

## Discussion

Previous studies have indicated that IV ketamine infusions can serve as an effective course of treatment for patients with TRD (23, 24). This study aimed to examine the safety and effectiveness of at-home use of SL ketamine for TRD and treatment-resistant anxiety. This study has shown that in as few as three doses of ketamine therapy nearly 50% of patients with moderate to severe depression saw an improvement of reducing their PHQ-9 and GAD-7 scores to half of their intake scores. This reduction rate improved to 60% in patients who completed a clinically recommended six ketamine RDT course of treatment. Of note, for patients with moderate to severe intake GAD-7 scores there was a clinical effect with ketamine therapy of reducing anxiety categorization in as few as three doses, and of those who were with moderate to severe intake GAD-7 scores who completed six ketamine RDT 65.4%, saw their GAD-7 scores reduced to 50% or more of their intake scores. Reductions in scores for those who had only received 3-RDT matched those who had completed 6-RDT (see Figure 2), and it is reasonable to speculate that there would be maintenance of improvement with three additional RDT ketamine treatments.

Our findings indicate that SL ketamine is clinically effective in reducing depression and anxiety in an at-home setting. The use of SL ketamine at home presents a promising approach in the treatment of TRD where treatment resistance may be the result of prior treatment approaches being mismatched with underlying neurobiological etiology. Since most antidepressants target monoaminergic mechanisms, the effectiveness of ketamine's pharmacological profile suggests an underlying role for glutamate in some individuals who experience TRD (47).

Limitations of the study were that it was a retrospective review of chart data from a self-selecting convenience sample. Measures of depression and anxiety relied extensively on self-report, though there was considerable provider care given. There was no placebo/control group, and given the dissociative effects of ketamine, a strong placebo effect is possible. Caution should be exercised whenever treating patients with depression and anxiety, especially those with complicating presenting symptoms (i.e., suicidality) or health problems (i.e., hypertension, substance abuse). While the majority of patients respond after 3 doses, we did not determine whether there are differences in the durability of response based on the number of treatments,; this an active area of investigation. Further work, which we are currently engaged in, will be needed to delineate not only the most effective dosing regimen, but also to identify likely responders based on patient characteristics and maintain continued analysis of patient data. The researchers also acknowledge the need for continued analysis of patient data. Nonetheless, we argue that SL ketamine offers a safe and effective tool in the treatment of anxiety and depression. This study represents a step toward consideration of at-home, SL ketamine for treatment-resistant depression and treatment-resistant anxiety.

In conclusion, this study adds to a growing literature on the effectiveness of ketamine treatment for TRD (22, 48). The improvements in depression and anxiety symptomology demonstrated in those who received as few as three at-home ketamine RDT present a safe, effective, and reasonable alternative to inpatient IV infusions of ketamine. These improvements in symptoms are further improved with completion of a clinically recommended course of six ketamine RDT. In addition to providing an effective treatment for those with TRD and comorbid anxiety, it also suggests alternative neurobiological modes for understanding MDD and anxiety symptoms. Additional studies including randomized trials, long-term impact on remission rates, combinations with additional psychosocial interventions, other functional outcomes, and cost-effectiveness analysis are needed.

## Data availability statement

The datasets presented in this article are not readily available because this data is a retrospective chart study of patient data from a private healthcare provider. Requests to access the datasets should be directed to kh@nue.life.

## Ethics statement

The studies involving human participants were reviewed and approved by Wheaton College, Wheaton, IL. The patients/participants provided their written informed consent to participate in this study.

## Author contributions

KH: project administration and writing—review and editing. WS: writing—review and editing and formal/statistical analysis. AS: data curation, review, and editing. PD: writing—review and editing. All authors contributed to the article and approved the submitted version.

## Conflict of interest

Authors KH, AS, and PD hold restricted shares of Nue Life Health, P.B.C. The remaining author declares that the research was conducted in the absence of any commercial or financial relationships that could be construed as a potential conflict of interest.

## Publisher's note

All claims expressed in this article are solely those of the authors and do not necessarily represent those of their affiliated organizations, or those of the publisher, the editors and the reviewers. Any product that may be evaluated in this article, or claim that may be made by its manufacturer, is not guaranteed or endorsed by the publisher.
